# Bioelectrical impedance vector analysis in older adults: reference standards from a cross-sectional study

**DOI:** 10.3389/fnut.2025.1640407

**Published:** 2025-07-23

**Authors:** Francesco Campa, Giuseppe Annunziata, Luigi Barrea, Alessandro Sampieri, Chiara Ceolin, Marina De Rui, Francesco Sguaizer, Cristian Petri, Fabrizio Spataro, Gabriele Mascherini, Margherita Micheletti Cremasco, Giuseppe Sergi, Tatiana Moro, Antonio Paoli

**Affiliations:** ^1^Department of Biomedical Sciences, University of Padua, Padua, Italy; ^2^Facoltà Di Scienze Umane, Della Formazione E Dello Sport, Università Telematica Pegaso, Naples, Italy; ^3^Dipartimento Di Psicologia E Scienze Della Salute, Università Telematica Pegaso, Naples, Italy; ^4^Geriatric Unit, Department of Medicine (DIMED), University of Padova, Padova, Italy; ^5^Department of Neurobiology, Care Sciences and Society, Karolinska Institutet and Stockholm University, Aging Research Center, Stockholm, Sweden; ^6^Department of Life Science and Systems Biology, University of Torino, Turin, Italy; ^7^Section of Physical Education and Sports, Department of Sports and Computer Science, Universidad Pablo de Olavide, Seville, Spain; ^8^Section of Clinical Nutrition and Nutrigenomics, Department of Biomedicine and Prevention, University of Rome Tor Vergata, Rome, Italy; ^9^Department of Experimental and Clinical Medicine, University of Florence, Florence, Italy

**Keywords:** body composition, BIA, BIVA, elderly people, R-Xc graph, phase angle

## Abstract

**Background and Aims:**

The bioelectrical impedance vector analysis (BIVA) requires population-specific references to correctly classify individuals based on body composition properties. The aim of this study was: (i) to develop new references specific to the older adult population; (ii) to evaluate vector patterns based on age and appendicular lean soft mass (ALMS); (iii) to compare the new references with others already existing in the literature.

**Methods:**

The present study included 835 older adults [472 women (mean age 73.9 ± 7.4 years, BMI 27.2 ± 5.4 kg/m^2^) and 363 men (mean age 73.1 ± 7.2 years, BMI 27.0 ± 4.4 kg/m^2^)]. Bioimpedance analysis was conducted using a phase-sensitive foot-to-hand technology at 50 kHz. Bioelectrical properties were analyzed among participants grouped by age categories and ALSM tertiles. New bivariate tolerance ellipses for resistance (R) and reactance (Xc), standardized by participants’ height (H), were compared with data from adult populations and the original BIVA references proposed by Piccoli in 1995 (ages 15–85).

**Results:**

New reference values for older adults were established. Significant differences (*p* < 0.001) in R/H and phase angle were observed when older adults were grouped by age categories, while R/H, Xc/H, and phase angle showed significant differences among ALSM/H^2^ tertiles. The mean bioelectrical vector for older adults differed from the references in the literature, showing a moderate magnitude relative to Piccoli’s original BIVA references (men: D^2^ = 0.6; women: D^2^ = 0.5) and a larger magnitude compared to the adult standards (men: D^2^ = 1.7; women: D^2^ = 1.8).

**Conclusion:**

This study provides BIVA references for older adults. Aging was associated with increased R/H and decreased phase angle, whereas older individuals with higher ALSM exhibited a greater phase angle and lower R/H, and Xc/H. The original BIVA references proposed in 1995 lack specificity and are no longer recommended for future use, as age-specific bioelectrical references are now available.

## Introduction

Bioelectrical impedance analysis (BIA) is a rapid, easy-to-use, and non-invasive method for evaluating body composition (1). The bioelectrical parameters of resistance (R) and reactance (Xc) can be incorporated into regression models developed against laboratory methods such as dual-energy X-ray absorptiometry, dilution techniques, or magnetic resonance imaging to estimate body mass components (2). However, the accuracy of these models depends on the specific characteristics of the populations to which they are applied. Accurate assessment body composition is achievable only when the selected equations are tailored to the target population (3).

In 1994, Piccoli et al. (4) highlighted the limitations of predictive equations and proposed an alternative approach based on the analysis of raw parameters. This method, known as bioelectrical impedance vector analysis (BIVA), involves combining R and Xc data into a vector representation on a Cartesian plane. To evaluate vector positioning, the R-Xc graph was subsequently integrated with tolerance ellipses that reflect the characteristics of the population from which they were derived (5). These ellipses, with a major longitudinal axis and a minor transverse axis, represent variations in fluid levels and their distribution between intracellular and extracellular compartments, respectively (6).

BIVA is widely used in both research and clinical practice to monitor bioelectrical measurements and infer hydration status and lean soft tissues without relying on the inherent estimation errors of predictive equations (6, 7). Over the years, technology- and population-specific reference ellipses have been developed to enhance BIVA’s applicability, covering both athletes and the general individuals (8). For the general population, tolerance ellipses are available for adolescents (9) and adults up to 65 years old (10), replacing the original ellipses proposed by Piccoli et al. (5) along with the BIVA’s inception. The limitations of Piccoli’s references, particularly the inclusion of individuals spanning diverse age groups (15 to over 80 years) along with the evolution of BIA-devices, have been highlighted by recent studies (10, 11), suggesting the need to use updated and population-specific references. However, tailored BIVA references for older adults aged 65 and above using phase-sensitive devices are currently lacking.

Aging leads to a deviation of the vector from the center of the adult ellipses, due to a decrease in intracellular water and an increase in extracellular water, primarily attributable to a reduction in lean soft mass (10–12). Although the adult population ellipses can be utilized to assess older adults by identifying a moderated physiological rightward shift of the vector within the R-Xc graph (10), having specific normality zones for older adults would provide a more appropriate assessment of their body composition. Furthermore, since recent research continues to rely on the original BIVA references (13, 14), additional evidence regarding their characteristics could help raise awareness about the importance of using updated and population-specific references.

Therefore, the aims of the present study were to: (i) develop population-specific reference ellipses for the general elderly population; (ii) to evaluate vector patterns based on age and appendicular lean soft mass; (iii) to compare the new references with others already existing in the literature.

## Methods

### Study design and participants

The present investigation was conceived as a multicenter, cross-sectional study. The study collected data at a national level from multiple cities across various Italian territories. Recruitment occurred through advertisements located in medical and sports center from January 2024 to January 2025. Subjects were offered the opportunity to receive a free assessment of their body composition. Exclusion criteria included the inability to collect bioelectrical measurements, pregnancy, being younger than 65 years, or having amputations or the presence of pacemakers that could interfere with bioimpedance outputs. The selection was also based on ensuring that at least 90% of the participants displayed a similar ethnicity (Caucasian).

A total of 835 older adults [472 women (mean age 73.9 ± 7.4 years, body mass index 27.2 ± 5.4 kg/m^2^) and 363 men (mean age 73.1 ± 7.2 years, body mass index 27.0 ± 4.4 kg/m^2^)] were included in the present study. After a detailed explanation of the procedures, the participants signed an informed consent. All research procedures were reviewed and approved by the Ethical Committee board of the University of Padova (approval code: HEC-DSB/02–2023) and were conform to the Declaration of Helsinki concerning studies involving human subjects.

### Procedures

All the bioelectrical impedance analyses were performed by using foot-to-hand phase sensitive impedance analyzers (BIA 101, 101 anniversary, or BIVA PRO, Akern, Pisa, Italy) at a single frequency of 50 kHz with 250 μA. Measurements were made on cots isolated from electrical conductors, the subjects were in the supine position with a leg opening of 45° compared to the median line of the body and the upper limbs, distant 30° from the trunk (15). After cleaning the skin with alcohol, four low intrinsic impedance adhesive electrodes (Biatrodes Akern, Pisa, Italy) were placed according to international guidelines (15). The participants were instructed to avoid any food of beverage for the previous four hours, as well as intensive exercise or alcohol intake for the previous 12 h before the test. Prior to each test session, the accuracy of the analyzer was validated using a reference circuit with acceptance for R measurements of 383 ohm (*Ω*) and Xc values of 46 Ω. The coefficient of variation values ranged from 0.2 to 0.4% for R and from 0.6 to 0.8% for Xc. The technical error of measurements ranged from 2.7 to 2.9 Ω and from 0.4 to 0.6 Ω for R and Xc, respectively.

ALSM was calculated using the predictive equations proposed by Sergi et al. (16) [ALSM (kg) = − 3.964 + 0.227 × H^2^/R + 0.095 × body mass + 1.384 × sex (0 for women and 1 for men) + 0.064 × Xc] and standardized by height squared.

Total body water was estimated for descriptive purposes using the Lukaski equation (17) as follows: Total body water (L) = 0.377 × H^2^/R + 0.14 × body mass – 0.08 × age + 2.9 × sex (0 for women and 1 for men) + 4.65.

### Statistical analysis

Statistical analysis was conducted using the BIVA software (18) and the JASP v. 0.19.1.0 program. A one-way analysis of variance (ANOVA) was performed to evaluate differences in R/H, Xc/H, phase angle, and body mass among participants grouped by age categories (65–69, 70–74, 75–79, and ≥80 years) and ALSM/H^2^ tertiles. Bonferroni correction was applied for post-hoc analyses. The two-sample Hotelling’s T^2^ test was used to compare the differences in the mean bioelectrical impedance vector between the reference values provided for the adult population (10) and those provided by Piccoli et al. (5) study. The Mahalanobis distance (D^2^), which represents a multivariate measure of effect and a multivariate measure of distance, was calculated to determine the magnitude of difference between the mean group vectors. D^2^ was interpreted according to the following Stevens (19) guidelines: 0.25–0.49: small; 0.5–0.99: medium; ≥1: large. Statistical significance was set at *p* < 0.05.

## Results

**Figure 1:**
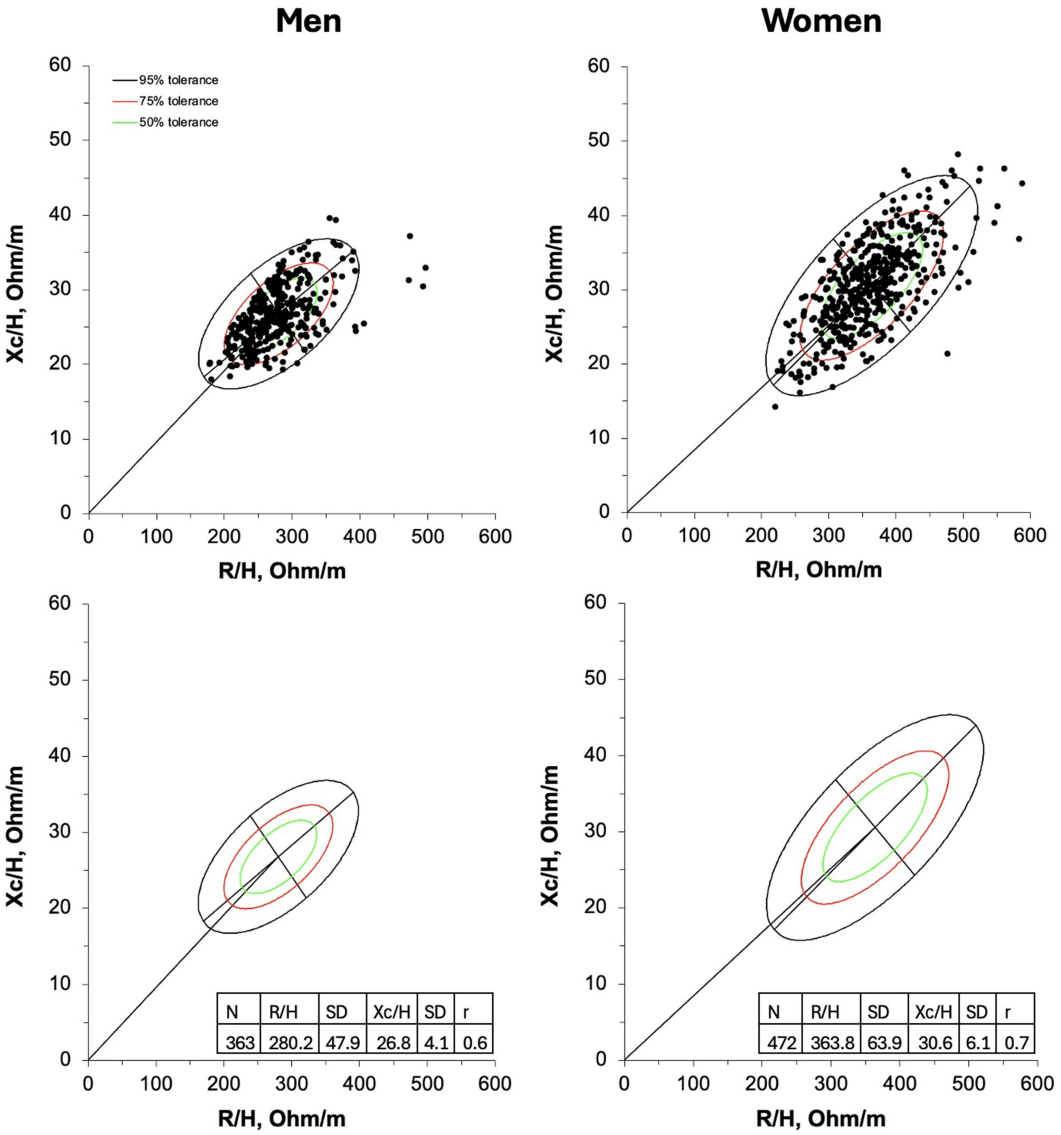
R-Xc graphs developed with bivariate resistance (R) and reactance (Xc) mean values standardized for the subjects’ stature (H) for men (on the left) and women (on the right). On the upper panel individual vectors are shown. On the lower panel, empty 50,75, and 95% tolerance ellipses are depicted with descriptive data; r, coefficient of correlation between R/H and Xc/H.

The new 50, 75, and 95% tolerance ellipses generated for male and female older adults are presented in Figure 1.

**Figure 2:**
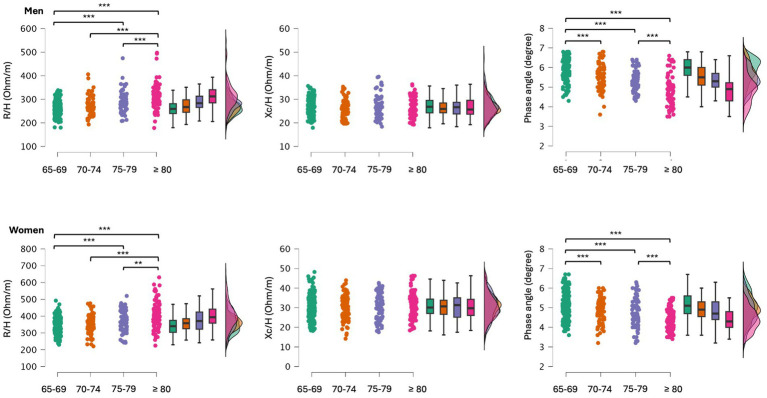
*Post-hoc* comparisons between older adults grouped by age categories. R/H, resistance standardized for subjects’ stature; Xc/H, reactance standardized for subjects’ stature; **p < 0.01; ***p < 0.001.

When participants were grouped for age categories significant differences were found for R/H (men: *F* = 29.4, *p* < 0.001; women: *F* = 25.8, *p* < 0.001) and PhA (men: *F* = 47.3, *p* < 0.001; women: *F* = 32.8, *p* < 0.001). Post-hoc comparisons are shown in Figure 2. Differences in body mass were also found between age categories (men: *F* = 22.8, *p* < 0.001; women: *F* = 24.8, *p* < 0.001), with participants aged ≥80 years having the lowest body mass, and those aged 75–79 being lighter than those aged 65–69. Descriptive statistics of the participants sorted by age groups are reported in Supplementary Table 1.

**Figure 3:**
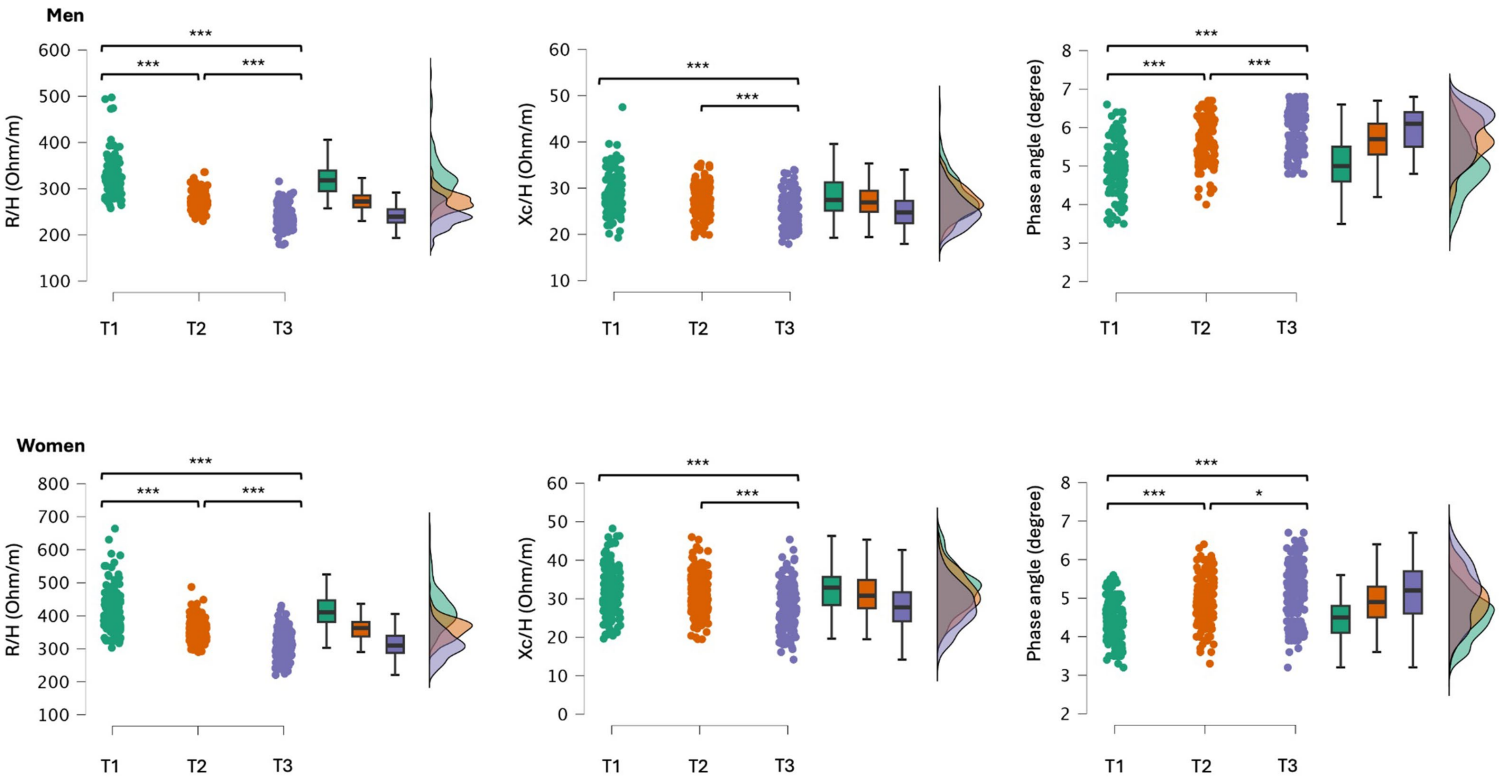
*Post-hoc* comparisons between older adults grouped by tertiles (T1, 1st tertile, T2, 2nd tertile and T3, 3rd tertile) according to their ALSM/H^2^; T1and T3 included the participants with lower and higher ALSM/H^2^, respectively. R/H, resistance standardized for subjects’ stature; Xc/H, reactance standardized for subjects’ stature; *p < 0.05; **p < 0.01; ***p < 0.001.

When participants were divided into tertiles based on ALSM/H^2^ levels, the first tertile comprised individuals with low ALSM/H^2^, with ages ranging from 65 to 94 for men and 65 to 97 for women. The second tertile included participants with average ALSM/H^2^, with ages ranging from 65 to 89 for men and 65 to 92 for women. Finally, the third tertile encompassed participants with high ALSM/H^2^, with ages ranging from 65 to 91 for men and 65 to 88 for women. Significant differences were found for R/H (men: *F* = 191.6, *p* < 0.001; women: *F* = 211.5, *p* < 0.001), Xc/H (men: *F* = 20.6, *p* < 0.001; women: *F* = 24.9, *p* < 0.001), and PhA (men: *F* = 71.2, *p* < 0.001; women: *F* = 45.6, *p* < 0.001). Post-hoc comparisons are shown in Figure 3. Differences in body mass were also observed (men: *F* = 140.2, *p* < 0.001; women: *F* = 366.9, *p* < 0.001), where a higher ALSM/H^2^ corresponded to a greater body mass. Descriptive statistics of the participants sorted by ALSM/H^2^ tertiles are reported in Supplementary Table 2.

**Figure 4:**
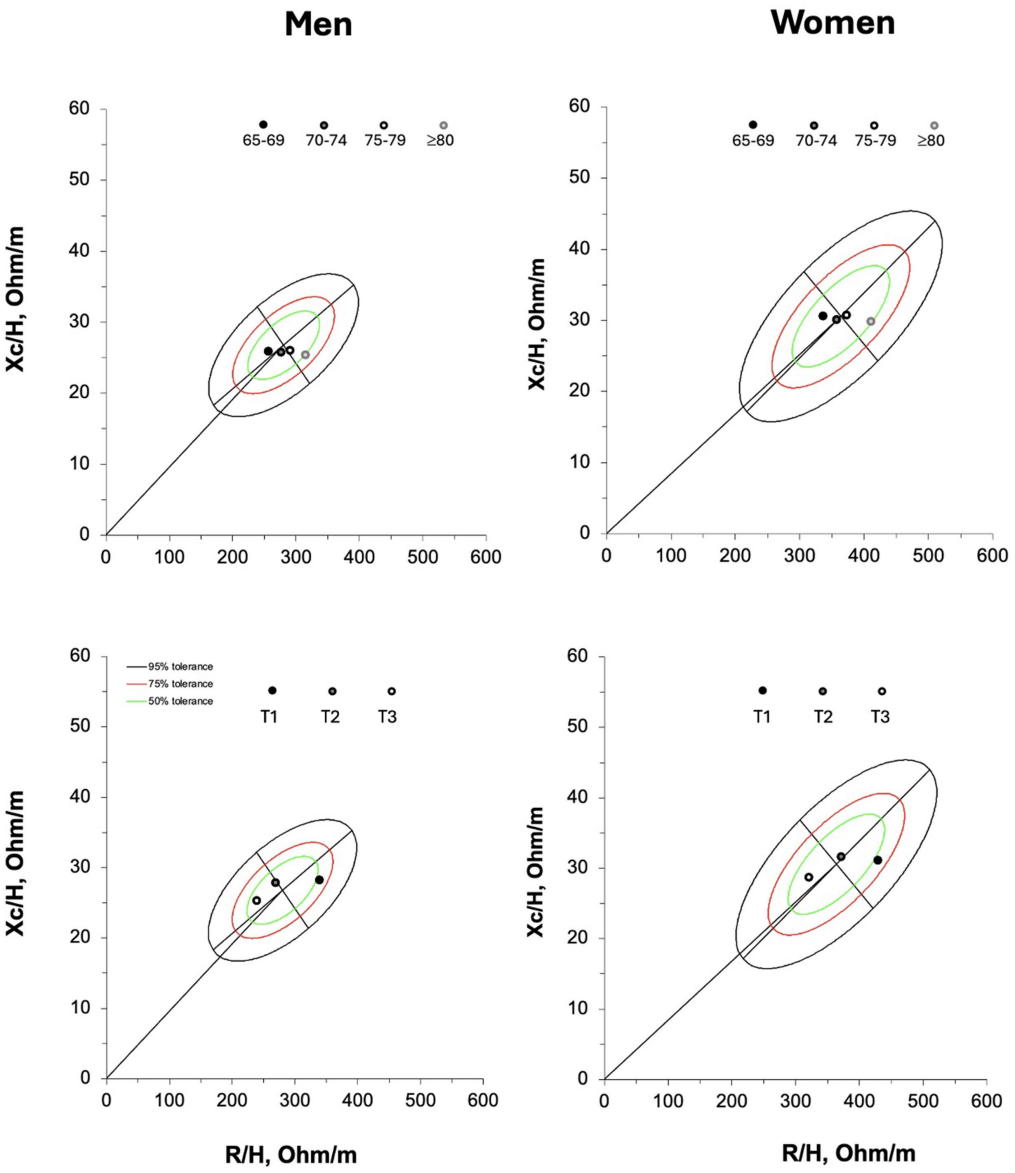
On the upper panel, the mean vectors of the participants grouped by age categories. R/H = resistance standardized for subjects’ stature; Xc/H, reactance standardized for subjects’ stature. On the lower panel, the mean vectors of the older adults grouped by tertiles (T1, first tertile, T2, second tertile and T3, third tertile) according to their appendicular lean soft mass standardized for subjects’ stature (ALSM/H^2^); T1and T3 included the participants with lower and higher amount of ALSM/H^2^, respectively.

Figure 4 graphically represents the vector distribution of the participants spitted by age groups and tertiles of ALSM/H^2^. A rightward vector displacement occurred from 65 to 69 to ≥80 years old male and female participants with vectors distributed within the 50% tolerance ellipse. The mean vector of the participants grouped in the first tertile (lower ALSM/H^2^) fall outside of the 50% tolerance ellipse at the right of the major axis, for both men and women. On the contrary, the mean vector of the participants included in the second and third tertiles were within the 50% tolerance ellipse at the left of the major axis.

**Figure 5:**
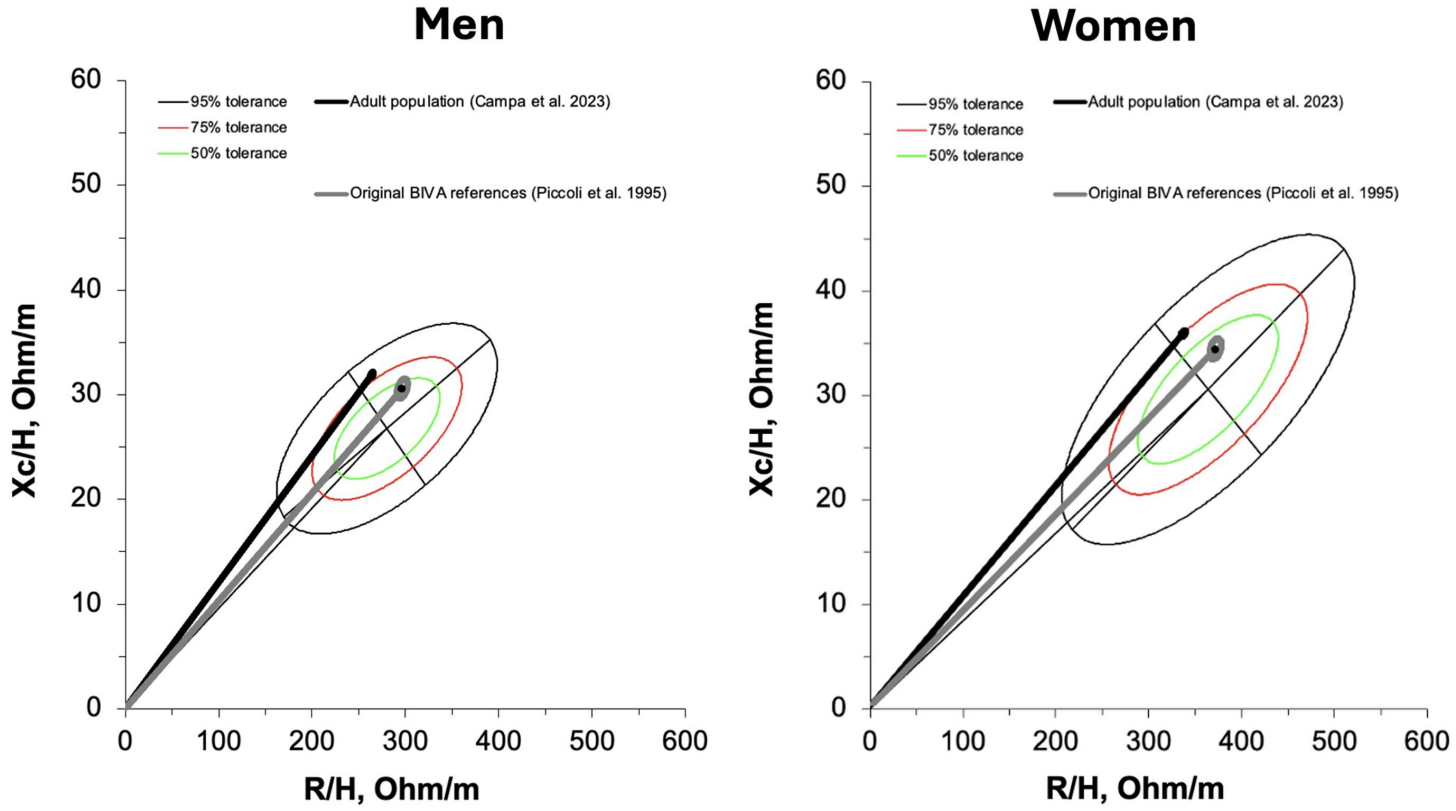
Comparison between bioelectrical reference data of adults (10) and original bioelectrical standards (5) with the new tolerance ellipses for the older adult population.

The comparison between the new references and other standards available in the literature revealed significant differences, although with a smaller magnitude (moderate vs. large effect size) when compared to the references proposed by Piccoli (5) (men: D^2^ = 1.7; women: D^2^ = 1.8) than to those derived from the adult population (10) (men: D^2^ = 0.6; women: D^2^ = 0.5). The mean vector of the population studied by Piccoli (5) fell within the 50% tolerance ellipse for both older males and females, whereas the mean vector of the adult population (10) was located outside the ellipse for both sexes, as shown in Figure 5.

## Discussion

The primary aim of the present study was to establish new bioelectrical reference values specifically designed for conducting BIVA with phase-sensitive foot-to-hand devices in the elderly population. A secondary objective was to analyze vector patterns based on age categories and ALSM/H^2^. Thirdly, the study sought to compare these new references with those of the adult population and also with the original references proposed alongside the invention of BIVA in 1995.

When participants were grouped by age categories, significant differences were found in R/H and phase angle. These differences resulted in a rightward shift of the vector within the R-Xc graph from the youngest to the oldest categories. The increase in R/H and the decrease in phase angle can primarily be attributed to reduced lean soft mass, particularly in the appendicular region, a condition typically associated with aging (20, 21). R/H tends to increase with age due to the loss of conductive lean soft mass, while Xc/H may remain relatively stable or decrease less markedly, since cell membrane integrity can be preserved longer or influenced by other factors like hydration or inflammation (22). This explains why aging vectors shift mainly to the right rather than along a diagonal line. Previous findings that examined the effects of sedentary behavior combined with aging showed a progressive decrease in Xc/H, in contrast to what occurs following resistance training and supervised dietary interventions (23). However, in these longitudinal studies, which lasted only a few months, an increase in body mass was also observed, an opposite trend to the decreasing body mass observed across the age groups in the present study. In our data, the <80 age group had the lowest average body mass, consistent with this trend. Notably, body mass loss in aging may involve both fat and fat-free mass, creating opposing effects on Xc: loss of intracellular water reduces cell mass and Xc, while reduced extracellular fluid from lower fat mass may increase Xc relative to body size (24). These opposing forces could explain the relatively stable Xc/H across age groups in our study, despite the clear upward trend in R/H and decline in phase angle. Weight loss is a common characteristic of aging after 65 years (25). Thus, the effect of aging on Xc could have been twofold, ultimately resulting in the absence of any noticeable change: on one hand, a reduction due to the loss of lean soft mass, and on the other, an increase in Xc as a consequence of body mass loss. Overall, the shift in the bioelectrical vector direction with age may reflects complex physiological changes, including alterations in hydration status, body composition, and cell health, and justifies the need for updated reference values in elderly populations.

The influence of body composition on bioelectrical parameters have been better explored by considering elderly participants divided into tertiles based on ALSM/H^2^. Particularly, older adults with higher ALSM/H^2^ demonstrated lower Xc/H and R/H values, along with a higher phase angle, with a vector positioned to the left of the major axis within the 50% tolerance ellipse. However, it is well established that during lean soft mass gain following resistance training, there is typically an increase in Xc/H accompanied by a decrease in R/H in older individuals (23). The findings of this study suggest that lower Xc/H does not necessarily correspond to lower ALSM. This observation is due to subjects with lower Xc/H but an elevated phase angle often exhibiting greater ALSM compared to those with higher Xc/H. This phenomenon may be attributed to higher body fat levels, which can reduce Xc due to potential water retention in tissues. This scenario likely reflects a condition where older adults adhering to an uncontrolled diet manage to preserve lean mass at the expense of optimal body composition (fat-to-muscle ratio). This contrasts with regulated dietary and training interventions, where higher lean soft mass is associated with increased Xc/H and decreased R/H (26). In these cases, the phase angle becomes a highly informative parameter. With this knowledge, a combined evaluation approach that considers both Xc and phase angle should be incorporated into the development of new predictive models. However, it is worth noting that Xc and phase angle, unlike R, exhibit significant variability across different technologies (27, 28), resulting in predictive models that are highly device-specific. BIVA, by enabling a combined assessment of bioelectrical properties, continues to be one of the most reliable approaches for analyzing bioelectrical data and assessing body composition.

As research continues to advance the development of bioelectrical-based predictive equations for estimating body composition, it also aims to provide increasingly specific BIVA references tailored to age, gender, and physical activity levels (29). In addition to the specificity related to the populations under study, lack of agreement among bioelectrical technologies currently necessitate that reference standards, as well as predictive equations, be as specific as possible to the technology used (27, 28, 30). In fact, the reference data from the present study appear to differ from those obtained using standing position technology (31), where the average phase angle values were apparently lower for the same age groups. Using foot-to-hand phase-sensitive devices, several tolerance ellipses have been proposed for the general population, including athletes, yet data on elderly individuals remain unavailable (8, 10). At the same time, the limitations of the pioneering ellipses proposed by Piccoli and collaborators (5) have become evident. While these ellipses enabled the use of BIVA for many years, their update has proven necessary, particularly in light of recent studies showing that their center aligns disproportionately with elderly individuals (10), as well as malnourished or sarcopenic subjects (11). The results of this study confirm that the former BIVA ellipses closely resemble the average data of the elderly population. This aspect could stem from several factors, including gaps in the sampling methodology used in Piccoli’s study (5), the effects of secular trends on body composition (32), and potential evolution or differences in the measurements obtained with newer BIA devices, whose outputs may differ from those of older instruments (33). As a result, the use of population-specific BIVA references is strongly recommended, particularly given their current availability. These updated references are essential to ensure accuracy and relevance in body composition assessment.

The strength of this study lies in the use of a phase-sensitive, single-frequency technology that remains one of the most widely used for conducting BIVA today (8). However, some limitations should be acknowledged. First, the results of this study cannot be generalized to other BIA technologies (e.g., standing-position multifrequency devices or foot-to-hand devices that are not phase-sensitive), as these may lack agreement with the instrument used in this study. Second, the participants in this study were recruited within the Italian territory, which means that potential differences in body composition due to geographic origin may emerge when applying these references to individuals from other countries or races. Future studies should investigate changes in BIVA patterns in response to physical exercise or dietary interventions in older adults. Furthermore, they should evaluate whether the newly proposed reference standards can accurately distinguish elderly individuals with conditions that affect body composition. Future studies should assess whether the newly proposed reference standards can effectively differentiate elderly individuals with conditions that impact body composition.

## Conclusion

This study offers updated bioelectrical reference values specifically tailored for older adults. It highlights that aging is characterized by increased R/H and decreased phase angle, while older individuals with higher ALSM may exhibit a greater phase angle alongside lower R/H and Xc/H. With the availability of age-specific bioelectrical references for key populations, generalized references such as those proposed by Piccoli should now be set aside. However, their valuable legacy in enabling the use of the BIVA method for decades is duly recognized.

## Data Availability

The raw data supporting the conclusions of this article will be made available by the authors, without undue reservation.
