# Correction: Bioelectrical impedance vector analysis in older adults: reference standards from a cross-sectional study

**DOI:** 10.3389/fnut.2025.1673638

**Published:** 2025-08-14

**Authors:** Francesco Campa, Giuseppe Annunziata, Luigi Barrea, Alessandro Sampieri, Chiara Ceolin, Marina De Rui, Francesco Sguaizer, Cristian Petri, Fabrizio Spataro, Gabriele Mascherini, Margherita Micheletti Cremasco, Giuseppe Sergi, Tatiana Moro, Antonio Paoli

**Affiliations:** ^1^Department of Biomedical Sciences, University of Padua, Padua, Italy; ^2^Facoltà Di Scienze Umane, Della Formazione E Dello Sport, Università Telematica Pegaso, Naples, Italy; ^3^Dipartimento Di Psicologia E Scienze Della Salute, Università Telematica Pegaso, Naples, Italy; ^4^Geriatric Unit, Department of Medicine (DIMED), University of Padova, Padova, Italy; ^5^Department of Neurobiology, Care Sciences and Society, Karolinska Institutet and Stockholm University, Aging Research Center, Stockholm, Sweden; ^6^Department of Life Science and Systems Biology, University of Torino, Turin, Italy; ^7^Section of Physical Education and Sports, Department of Sports and Computer Science, Universidad Pablo de Olavide, Seville, Spain; ^8^Section of Clinical Nutrition and Nutrigenomics, Department of Biomedicine and Prevention, University of Rome Tor Vergata, Rome, Italy; ^9^Department of Experimental and Clinical Medicine, University of Florence, Florence, Italy

**Keywords:** body composition, BIA, BIVA, elderly people, R-Xc graph, phase angle

In the published article, there was an error in the **Affiliations**. Instead of “^2^Department of Pharmacy, University of Naples “Federico II”, Naples, Italy” and “^3^Dipartimento di Medicina Clinica e Chirurgia, Unit of Endocrinology, Federico II University Medical School of Naples, Naples, Italy”. Affiliation 2 should have been written as “Facoltà Di Scienze Umane, Della Formazione E Dello Sport, Università Telematica Pegaso, Naples, Italy”. Affiliation 3 should have been written as “Dipartimento Di Psicologia E Scienze Della Salute, Università Telematica Pegaso, Naples, Italy.”

The original article has been updated.

