# Supplementary material for: Bioelectrical impedance vector analysis in older adults: reference standards from a cross-sectional study
Source: Front Nutr. 2025 Jul 23;12:1640407. doi: 10.3389/fnut.2025.1640407 (PMC12325078; doi:10.3389/fnut.2025.1640407)
Supplement: Supplementary file 1 [file Table_1.docx]

| **Supplementary table 1**. Desctiptive characteristics (mean ± SD) for the participants grouped by age categories. | | | | | | | | |
| --- | --- | --- | --- | --- | --- | --- | --- | --- |
|  | 65-69 years | | 70-74 years | | 75-79 years | | ≥ 80 years | |
|  | Men (N=149) | Women (N=184) | Men (N=73) | Women (N=99) | Men (N=69) | Women (N=81) | Men (N=72) | Women (N=108) |
| Age (y) | 66.5 ± 1.4 | 67.1 ± 1.3 | 71.9 ± 1.4 | 72.0 ± 1.4 | 76.7 ± 1.4 | 77.1 ± 1.3 | 84.7 ± 3.6 | 85.1 ± 3.9 |
| Stature (m) | 173.3 ± 7.6 | 159.5 ± 7.1 | 171.8 ± 6.4 | 156.5 ± 6-6 | 169.4 ± 7.8 | 154.9 ± 5.9 | 164.5 ± 7.8 | 153.1 ± 7.2 |
| Body mass (kg) | 84.7 ± 15.6 | 71.6 ± 14.1 | 79.3 ± 13.1 | 69.1 ± 15.8 | 76.7 ± 12.8 | 65.6 ± 12.7 | 68.9 ± 14.5 | 57.5 ± 12.1 |
| ALSM/H^2^ (kg/m^2^) | 7.9 ± 0.8 | 6.6 ± 0.9 | 7.6 ± 0.8 | 6.5 ± 0.9 | 7.4 ± 0.9 | 6.3 ± 0.9 | 6.9 ± 1.1 | 5.7 ± 0.8 |
| TBW (l) | 39.5 ± 5.3 | 27.3 ± 4.6 | 37.1 ± 5.3 | 25.6 ± 4.9 | 34.7 ± 5.1 | 23.9 ± 4.9 | 30.5 ± 5.9 | 20.7 ± 4.4 |
| Abbreviations: ALSM/H^2^= Appendicular lean soft mass in kg standardized for subjects’ stature in meters; TBW= total body water. | | | | | | | | |
