# Supplementary material for: Bioelectrical impedance vector analysis in older adults: reference standards from a cross-sectional study
Source: Front Nutr. 2025 Jul 23;12:1640407. doi: 10.3389/fnut.2025.1640407 (PMC12325078; doi:10.3389/fnut.2025.1640407)
Supplement: Supplementary file 2 [file Table_2.docx]

| **Supplementary table 2**. Desctiptive characteristics (mean ± SD) for the participants grouped by tertiles (T) of ALSM/H^2 .^ | | | | | | |
| --- | --- | --- | --- | --- | --- | --- |
|  | Lower ALSM/H^2^ (first T) | | Average ALSM/H^2^ (second T) | | High ALSM/H^2^ (third T) | |
|  | Men | Women | Men | Women | Men | Women |
| Age (y) | 77.7 ± 7.6 | 77.8 ± 8.1 | 71.1 ± 5.5 | 73.4 ± 6.5 | 70.6 ± 6.0 | 70.6 ± 5.4 |
| Stature (m) | 168.8 ± 8.5 | 155.0 ± 7.8 | 171.5 ± 7.7 | 156.9 ± 6.4 | 171.2 ±7.9 | 158.0 ± 7.3 |
| Body mass (kg) | 66.1 ± 10.2 | 53.9 ± 7.8 | 78.8 ± 9.1 | 64.5 ± 6.7 | 91.4 ± 15.1 | 82.0 ± 12.4 |
| ALSM/H^2^ (kg/m^2^) | 6.6 ± 0.5 | 5.4 ± 0.4 | 7.5 ± 0.2 | 6.2 ± 0.2 | 8.6 ± 0.6 | 7.4 ± 3.6 |
| TBW (l) | 30.6 ± 4.3 | 20.2 ± 3.4 | 36.7 ± 3.3 | 24.4 ± 2.6 | 41.8 ± 5.2 | 30.0 ± 4.4 |
| Abbreviations: ALSM/H^2^= Appendicular lean soft mass in kg standardized for subjects’ stature in meters; TBW= total body water. | | | | | | |
